# Housing tenure and early-life acute lower respiratory tract infection admissions in two national Scottish birth cohorts

**DOI:** 10.1136/bmjph-2024-001965

**Published:** 2025-07-17

**Authors:** Caroline Hart, Samantha Hajna, Bianca De Stavola, Ai Milojevic, Steve Cunningham, Jonathon Taylor, Pia Hardelid

**Affiliations:** 1 University College London, London, UK; 2 Population Policy and Practice, UCL GOS Institute of Child Health, London, UK; 3 Department of Health Sciences, Brock University, St Catharines, Ontario, Canada; 4 Social and Environmental Health Research, London School of Hygiene and Tropical Medicine, London, UK; 5 Department of Child Life and Health, Centre for Inflammation Research, Univerisity of Edinburgh, Edinburgh, UK; 6 Department of Civil Engineering, Tampere University, Tampere, Pirkanmaa, Finland; 7 Centre for Paediatric Epidemiology and Biostatistics, UCL GOSH PPP, London, UK

**Keywords:** Epidemiologic Factors, Sociodemographic Factors, Public Health

## Abstract

**Introduction:**

Early-life acute lower respiratory tract infections (LRTIs) have been associated with subsequent wheezing, asthma and mortality. Despite infants and young children spending much of their time at home, the association of housing circumstances, including housing ownership status, on acute LRTI hospital admissions is not well explored.

**Objective:**

To assess the association between housing tenure and the odds of hospital admission for acute LRTIs in children aged <2 years in Scotland.

**Methods:**

Scottish birth records were linked to maternal census data (2001 and 2011) to construct two birth cohorts: cohort 1 (C1; born 2000–2002) and cohort 2 (C2; 2010–2012). Linkage to hospital records provided information on acute LRTI admissions. Using multivariable logistic regression models, we estimated the association of housing tenure with the odds of ≥1 hospital admission for LRTI before the second birthday with adjustment for area of residence (urban/rural), maternal highest qualification level and maternal age.

**Results:**

There were 14 833 LRTI admissions in 12 527 children across both cohorts. 4.0% and 5.3% children in C1 and C2, respectively, had ≥1 LRTI admission. Compared with living in owned housing, the odds of LRTI admission were higher in children living in social rented housing (C1: OR 1.40, 95% CI 1.31 to 1.49; C2: 1.23, 1.16 to 1.31), private rented (C1: 1.24, 1.11 to 1.39; C2: 1.14, 1.06 to 1.21) and rent-free housing (C1: 1.53, 1.35 to 1.74; C2: 1.04, 0.80 to 1.36).

**Conclusion:**

Children living in rented housing had higher odds of early-childhood LRTI admission compared with those living in owned housing in Scotland. Further linkage to residential-level data could inform the design of LRTI prevention policies.

**WHAT IS ALREADY KNOWN ON THIS TOPIC**

- Early-life lower respiratory tract infection (LRTI) is associated with wheezing and asthma in childhood and respiratory disease in adulthood.
- Risk of hospitalisation for LRTI is higher among children from low-income neighbourhoods.
- Less is known about how housing ownership status contributes to these inequalities.

**WHAT THIS STUDY ADDS**

- Children living in social and private rented housing (compared with owned housing) have a higher risk of LRTI hospital admission.
- This study demonstrates the value of linking maternal Census and administrative health data in improving our understanding of how housing factors impact children’s respiratory health.

**HOW THIS STUDY MIGHT AFFECT RESEARCH, PRACTICE OR POLICY**

- Children living in rental housing are most affected by LRTI hospitalisations and could benefit from LRTI-lowering interventions.
- More research is needed to elucidate the mechanisms underlying the association between housing tenure and early-life LRTI admission.

##  Introduction

Acute lower respiratory tract infections (LRTIs) are a major child public health concern.^1^ They are a leading cause of hospital admissions and deaths in children <5 years old globally, with an estimated 40 million cases and 650 000 deaths in 2019 alone.^2^ In the UK, hospital admissions for LRTIs among young children were rising prior to the emergence of COVID-19.^3 4^ A growing body of evidence indicates that early-life LRTIs may have lasting effects on the developing lungs; LRTIs in infancy have been associated with chronic wheezing and asthma in later childhood and adulthood,^5^,^7^ and mortality in later life.^8 9^ These long-term significant health impacts underscore the need to safeguard lung health and minimise LRTI occurrence in early childhood.

Socio-economic deprivation has been associated with higher rates of paediatric LRTI-related admissions,^2 10^ with evidence indicating a recent widening of these disparities in the UK.^11^ The complex reasons behind these widening disparities remain poorly understood. A reliance on area-level indicators of social deprivation may have hindered the identification of the specific social and environmental factors influencing LRTI admission risk.^12^ Despite children spending approximately 90% of their time at home,^13^ LRTI research has focused largely on individual/familial risk factors, rather than on the wider determinants of health, such as housing circumstances.^14^ Children from lower-income households are more likely to reside in poor-quality and hazardous housing, characterised by overcrowding, dampness and cold.^15^ These adverse living conditions, which are more common in rental accommodation in the UK,^16^ have also been associated with respiratory symptoms.^17 18^

The impact housing ownership status has on child health represents an underexplored avenue through which social and environmental disparities can contribute to health inequalities. During the last two decades, many European countries have experienced a reduction in the supply of social housing combined with rising rental costs.^19^ Where there is a lack of regulation and enforcement of housing standards, it is the growing number of families in rental accommodation who are most likely to experience overcrowded, non-decent and unaffordable homes.^19^ In countries where no-fault evictions still exist, families living in private rental accommodation can feel unable to escalate repair and maintenance issues for fear of becoming homeless.^20^

This research aimed to investigate the impacts of housing tenure status on early-childhood LRTI admissions using linked Scottish administrative data. Specific objectives included (1) quantifying the burden of acute LRTI hospital admissions in two Scottish national birth cohorts, (2) describing differences in their socio-demographic circumstances and (3) estimating the association between housing tenure and LRTI hospital admissions in children aged <2 years. The output of this research may have policy implications for upstream housing interventions to reduce possible inequalities in LRTI admissions.

## Methods

### Data sources

We used seven administrative data sets (online supplemental table 1), including birth and death registrations, address records, maternity records (Scottish Morbidity Record; SMR-02), hospital admissions (SMR-01) and decennial Scottish Census data from 2001 and 2011. These de-identified data were linked using the Community Health Index (CHI) number, a unique patient identifier which enables the linkage of Scottish healthcare records from birth onwards.^21^ Using an existing national cohort for Scotland, developed for the ‘PICNIC’ study,^22^ we constructed two birth cohorts. The National Records for Scotland (NRS), which contained birth records, formed the cohorts' spine. The completeness of linkage between birth registration records and maternal and child CHI records was high at (99.8%) and (99.9%), respectively (personal email and linkage report, prepared by the NRS indexing team).

### Study population

We included all singleton children born in Scotland to Scottish resident mothers between 1 January 2000 and 31 December 2002, cohort 1 (C1), and 1 January 2010 to 31 December 2012, cohort 2 (C2). Children were followed up until their second birthday. Children who could not be linked to maternal Census records or who did not live in private households were excluded as we would have been unable to assign the main exposure to these children. To avoid the inclusion of stillbirths, births recorded as <24 weeks’ gestation or with a birth weight <500 g were excluded, as were children who died or emigrated before their second birthday.

### Measures

#### Outcome

Our outcome was defined as LRTI admission (yes/no), where each child was identified as having either one or more occurrences of acute LRTI admission, or no acute LRTI admission by their second birthday. We identified hospital admissions with a primary diagnosis of acute LRTI using the International Classification of Diseases diagnostic code, 10th edition (ICD-10). ICD-10 codes were grouped into five types: influenza (J10-J11), pneumonia (J12-J18), bronchitis (J20), bronchiolitis (J21) and unspecified (J22). Multiple records of admissions within the same child were collapsed into a single continuous in-person stay if the number of days between the discharge date of one admission and the subsequent admission was less than 1 day. Duplicate records, containing the same admission and discharge dates, for the same child were removed.

To calculate the total burden of hospital admissions attributable to any acute LRTI, we extracted all hospital admission events with a primary diagnosis of LRTI occurring between 1 January 2000 and 31 December 2004 and 1 January 2010 and 31 December 2014, where the patient was less than 2 years old. For the binary outcome of one or more LRTI admissions, we included the first recorded LRTI admission.

#### Exposure

Our exposure of interest was housing tenure which was derived from the 2001 and 2011 maternal Census records and determined using the following questions: *Does your household own or rent this accommodation?* and *Who is your landlord?* Housing tenure was categorised as owned (shared ownership/owned outright/owned with a mortgage), social rented (local council/housing association/registered social landlord), private rented (private landlord/letting agency) and rent-free (living in the house of an employer, family member or ‘other’).

### Covariates

The study population was described by the following characteristics: child (sex recorded at birth, parity, birth weight, estimated gestational age), maternal (country of origin, age in years, highest qualification level, history of smoking in pregnancy) and residential (area of residence, accommodation type, central heating type). The variables included in the statistical models were coded as follows: maternal age, obtained from the maternity/delivery records (<20, 20–29, 30–39, 40+years), highest maternal qualification level, derived from the Census (no qualifications, level 1—standard or equivalent, level 2, level 3, level 4—first or higher degree, not applicable (<16 years old) and area of residence, derived from the urban/rural classification (UR8),^23^ from birth registration records (large urban areas, other urban areas, accessible small towns, remote small towns, very remote small towns, accessible rural areas, remote rural areas, very remote rural areas); for the latter, we used the UR8 version closest to the children’s birth years (2003–2004) for births between 2000 and 2002; 2011–2012 for births between 2010 and 2012). We also considered the following variables as potential effect modifiers of the association between housing tenure and LRTI admission: parity (0, 1, ≥2 completed pregnancies) and smoking during pregnancy (yes/no).

### Statistical methods

We compared the characteristics of the children excluded from the study population because of missing maternal Census records to those included. To quantify the burden of acute LRTI hospital admissions in both cohorts (objective 1), we charted the frequency of LRTI admission types across quarters. To understand the association of housing tenure with socio-economic circumstances (objective 2), we examined how the distribution of child and family characteristics varied within each housing tenure category and cohort. We used logistic regression models to assess the association between housing tenure and LRTI admissions (objective 3) with the ‘owned’ tenure category representing the reference group, with/without adjustment for major covariates. Results were expressed as crude and adjusted ORs with 95% CIs, calculated using robust SEs to account for the clustering of children within families (approximately 8% of children in each cohort had the same mother and were therefore siblings). A graphical representation informed by a literature review was used to select the adjustment variables. We identified the area of residence (urban/rural), maternal age and maternal highest qualification as relevant adjustment covariates (online supplemental figure 1). In multivariable models, we sequentially added each of these adjustment covariates one by one and compared them with an empty model to assess how the coefficient estimates changed prior to including all three in one ‘maximally adjusted’ model. Only children with complete data for all three covariates were included in the regression models since missing values for the confounders were infrequent (3–4%). To assess the impact of potential selection bias, due to the exclusion of records with missing covariate values, we compared the baseline characteristics of those with/without complete records.

Evidence of differential associations by cohort was assessed by fitting the maximally adjusted model on the merged cohorts with an interaction term between housing tenure and binary variable indicating the cohort the record belonged to. We tested its significance using the likelihood ratio test (LRT). We further tested the potential effect modification of the association between housing tenure and LRTI admission due to parity and maternal smoking, again using the LRT, in each case with p values <0.05 taken as evidence of effect modification. All analyses were performed in the Scottish Safe Haven using STATA/MP 16.1 software.

### Patient and public involvement

Although we have not asked parents specifically about this aspect of the study, the study team has consulted several groups of parents about this project, including the Great Ormond Street Hospital Parents and Carers Advisory Group, and via charities including Shelter and National Children’s Bureau, regarding linkage of health and environmental data.

## Results

**Figure 1:**
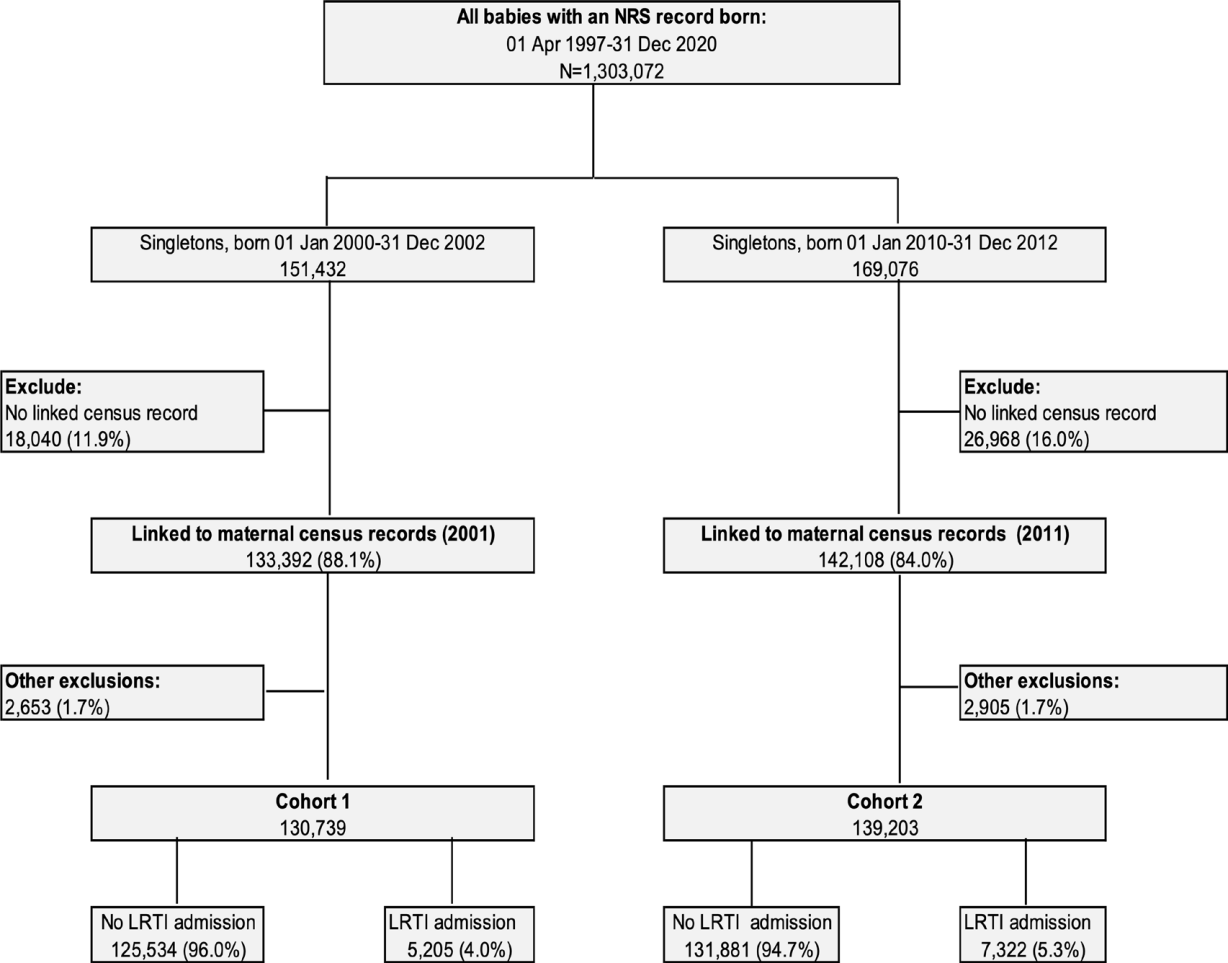
Flow chart of the derivation of study birth cohorts. LRTI, lower respiratory tract infection; NRS, National Records for Scotland.

Figure 1 outlines the selection of study participants. The proportion of children who could not be linked to a maternal Census record was higher in C2 (16% vs 12%). Other exclusions (C1: 2653 (1.7%), C2: 2905 (1.7%)) included birth weight <500 g, gestational age <24 weeks, mother not resident in Scotland, children living in communal dwelling (not a household) and children who had emigrated or died. After applying all exclusions, cohort 1 comprised 130 739 children, (born 2000–2002) and cohort 2 comprised 139 203 children (born 2010–2012). A total of 5205 children (4.0%) and 7322 (5.3%) in cohorts 1 and 2, respectively, had one or more LRTI admissions.

The distribution of children’s characteristics was broadly similar across the two cohorts (online supplemental table 2). Some notable differences included a twofold increase in mothers of non-UK origin (C1: 6.1% vs C2: 12.8%), fewer teenage pregnancies (C1: 7.5%; C2: 5.5%), a substantial increase in mothers achieving a first or higher degree (C1: 23.3%; C2: 36.9%) and a reduction in maternal smoking in pregnancy (C1: 23.7%; C2: 16.0%). In both cohorts (online supplemental table 3), the children excluded due to missing Census information were slightly more likely to have an LRTI admission (C1: 4.0% vs 4.4% and C2: 5.2 vs 5.7%). They were also more likely to have some characteristics associated with LRTI admission, such as low birth weight, preterm birth, more than one older sibling or a younger mother.

**Figure 2:**
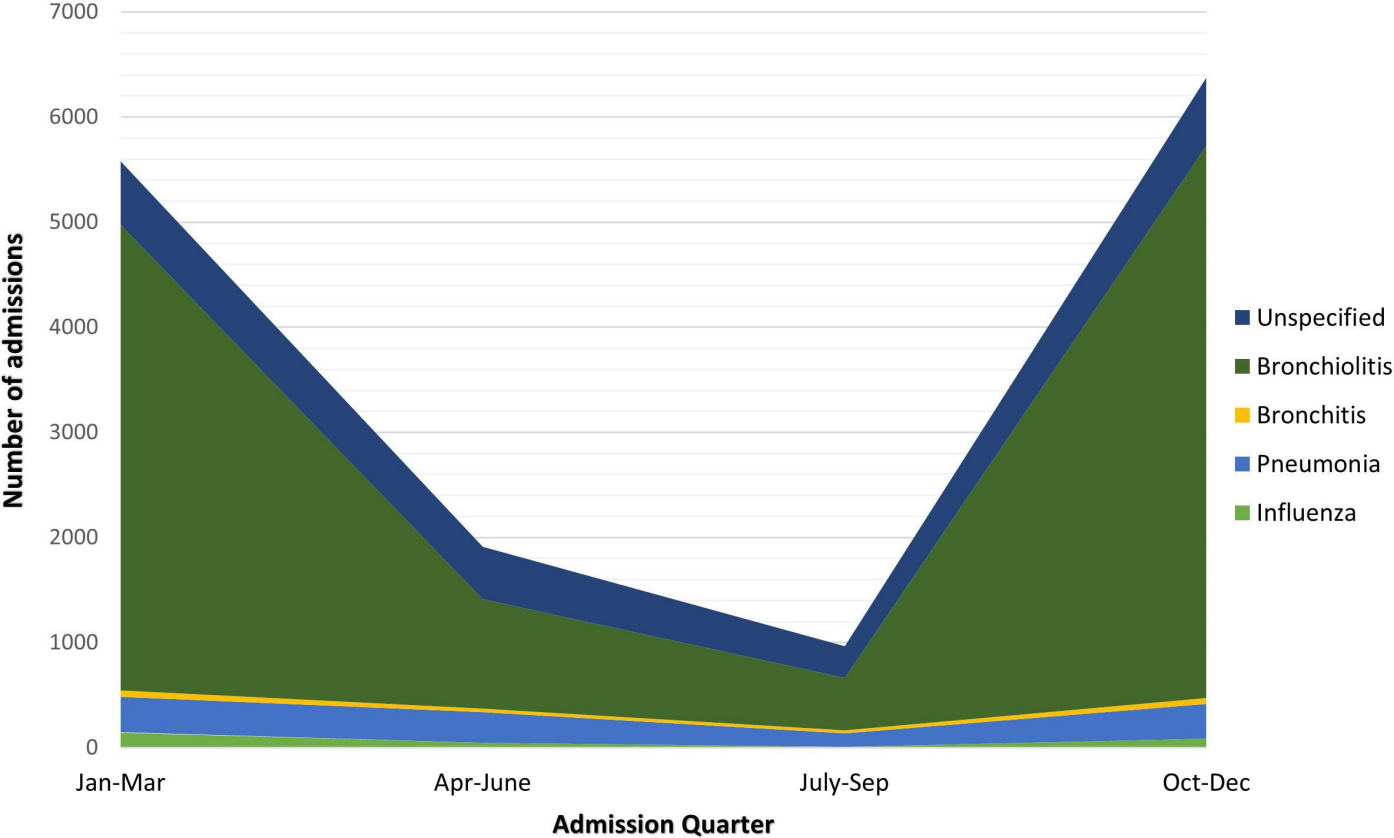
Number of lower respiratory tract infection admissions by type and quarter across the two cohorts (n=14 833). Monthly counts were aggregated to quarters due to small values.

Overall, across the two cohorts, there were 14 833 LRTI admissions in 12 527 children (with 10 832 children, 86.5%, having one LRTI admission and 1695, 13.5%, having >1 LRTI admission). Bronchiolitis was the most frequently coded LRTI, accounting for 75.6% (11 218) of all LRTI admissions. The burden of LRTI admissions was greater during the winter months (figure 2).

### Child and family characteristics stratified according to housing tenure

Most children lived in owned (C1: 64.5%; C2: 57.0%), followed by social rented accommodation (C1: 25.0%; C2: 24.4%) (table 1). The proportion of children living in private rental accommodation was almost three times higher in C2 (17.7% vs 6.5% in C1). LRTI admission was least frequent in owner-occupied households (C1: 3.4%; C2: 4.7%). In C1, LRTI admission was most common among rent-free residents (5.6%), followed by social renters (5.2%). In C2, it was most common among social renters (6.4%), followed by private renters (5.6%). In both cohorts, children living in social and private rented households, compared with owner-occupied, also experienced further LRTI risk factors, including >1 older sibling (see online supplemental table 4), low birth weight, preterm birth, young maternal age and low maternal qualifications.

**Table 1:** Child and family characteristics stratified by housing tenure (based on a subsample with complete records for all the adjustment variables)

	Cohort 1Births 2000–2002 (n=126 187)				Cohort 2Births 2010–2012 (n=135 646)			
	Owned	Social rent	Private rent	Rent-free	Owned	Social rent	Private rent	Rent-free
	N (%)	N (%)	N (%)	N (%)	N (%)	N (%)	N (%)	N (%)
All	81 451 (64.5)	31 569 (25.0)	8173 (6.5)	4994 (4.0)	77 312 (57.0)	33 064 (24.4)	24 011 (17.7)	1259 (0.9)
Lower respiratory tract infection admission	2775 (3.4)	1643 (5.2)	360 (4.4)	281 (5.6)	3609 (4.7)	2111 (6.4)	1350 (5.6)	63 (5.0)
*Child*								
Birth weight (g)								
<2500	3380 (4.1)	2406 (7.6)	469 (5.7)	379 (7.6)	2797 (3.6)	2219 (6.7)	1169 (4.9)	46 (3.7)
2500–3499	39 247 (48.2)	17 428 (55.2)	4331 (53.0)	2700 (54.1)	35 555 (46.0)	17 455 (52.8)	11 978 (49.9)	563 (44.7)
3500+	38 791 (47.6)	11 724 (37.1)	3369 (41.2)	1912 (38.3)	38 928 (50.3)	13 378 (40.5)	10 852 (45.2)	650 (51.6)
Missing	33 (0.1)	11 (0.1)	4 (0.1)	3.0 (0.1)	32 (0.1)	12 (<0.1)	12 (<0.1)	0 (0.0)
Gestational age (weeks)								
Preterm <37	4075 (5.0)	2200 (7.0)	475 (5.8)	348 (7.0)	3689 (4.7)	2146 (6.5)	1293 (5.4)	49 (3.9)
Term 37–41	55 620 (68.3)	21 500 (68.1)	5538 (67.8)	3464 (69.4)	53 240 (68.9)	23 192 (70.1)	16 479 (68.6)	866 (68.8)
Post-term 41+	21 736 (26.7)	7856 (24.9)	2158 (26.4)	1178 (23.6)	20 338 (26.3)	7705 (23.3)	6226 (25.9)	344 (27.3)
Missing	20 (0.1)	13 (<0.1)	2 (<0.1)	4.0 (0.1)	45 (0.1)	21 (0.1)	(0.1)	0 (0.0)
*Maternal*								
Maternal age (years)								
<20	2765 (3.4)	5129 (16.2)	847 (10.3)	1009 (20.2)	1803 (2.3)	4227 (12.8)	1517 (6.3)	49 (3.9)
20–29	29 816 (36.6)	17 352 (55.0)	4223 (51.7)	2649 (53.0)	25 511 (33.0)	19 523 (59.0)	14 280 (59.5)	617 (49.0)
30–39	46 474 (57.1)	8590 (27.2)	2941 (36.0)	1264 (25.3)	45 998 (59.5)	8660 (26.2)	7737 (32.2)	550 (43.7)
>40	2396 (2.9)	498 (1.6)	162 (2.0)	72 (1.5)	4000 (5.2)	654 (2.0)	477 (2.0)	43 (3.4)
Missing	0 (0.0)	0 (0.0)	(0.0)	0 (0.0)	0 (0.0)	0 (0.0)	0 (0.0)	0 (0.0)
Highest qualification								
No qualifications/not applicable*	5637 (6.9)	9596 (30.4)	1442 (17.7)	1568 (31.4)	1975 (2.5)	6638 (20.1)	2417 (10.1)	102 (8.1)
Level 1 standard	24 310 (29.9)	14 908 (47.2)	2954 (36.1)	2177 (43.6)	13 574 (17.6)	15 504 (46.9)	7587 (31.6)	251 (19.9)
Level 2 higher/advanced	16 326 (20.0)	3921 (12.4)	1343 (16.4)	540 (10.8)	11,278 (14.6)	4246 (12.8)	3390 (14.1)	202 (16.1)
Level 3 SVQ level 4	9227 (11.3)	2015 (6.4)	706 (8.6)	258 (5.2)	11 453 (14.8)	3723 (11.3)	3338 (13.9)	174 (13.8)
Level 4 first/higher degree	25 951 (31.9)	1129 (3.6)	1728 (21.2)	451 (9.0)	39 032 (50.5)	2953 (8.9)	7279 (30.3)	530 (42.1)
Missing	0 (0.0)	0 (0.0)	(0.0)	0 (0.0)	0 (0.0)	0 (0.0)	0 (0.0)	0 (0.0)
Country of origin								
UK-born	76 147 (93.5)	30 537 (96.7)	7179 (87.8)	4741 (94.9)	69 250 (89.6)	29 681 (89.8)	18 421 (76.7)	998 (79.3)
Non-UK-born	5303 (6.5)	1032 (3.3)	993 (12.2)	253 (5.1)	8056 (10.4)	3381 (10.2)	5588 (23.3)	261 (20.7)
Missing	1 (<0.1)	0 (0.0)	1 (<0.1)	0 (0.0)	6 (<0.1)	2 (<0.1)	2 (<0.1)	0 (0.0)
Tobacco smoking(during pregnancy)								
No	67 018 (82.3)	15 077 (47.8)	5299 (64.8)	2412 (48.3)	67 680 (87.5)	19 156 (57.9)	17 844 (74.3)	1049 (83.3)
Yes	11 400 (14.0)	14 760 (46.7)	2534 (31.0)	2279 (45.6)	5472 (7.1)	11 863 (35.9)	4763 (19.8)	126 (10.0)
Missing	3033 (3.7)	1732 (5.5)	340 (4.2)	303 (6.1)	4160 (5.4)	2045 (6.2)	1404 (5.9)	84 (6.7)
*Residential*								
Area of residence urban/rural								
Urban	66 356 (81.5)	27 939 (88.5)	5809 (71.0)	3936 (78.8)	64 209 (83.1)	29 567 (89.4)	20 449 (85.2)	604 (48.0)
Rural	15 095 (18.5)	3630 (11.5)	2364 (29.0)	1058 (21.2)	13 103 (16.9)	3497 (10.6)	3562 (14.8)	655 (52.0)
Missing	0 (0.0)	0 (0.0)	0 (0.0)	0 (0.0)	0 (0.0)	0 (0.0)	0 (0.0)	0 (0.0)

Percentages may not add up to 100% due to rounding.

* The not applicable group was combined with the ‘no qualifications’ group due to small values.

SVQ, Scottish Vocational Qualification.

### LRTI admissions and housing tenure

We identified statistically significant effect modification of housing tenure on LRTI admission odds by cohort (LRT, p<0.001). We therefore reported the results for each cohort separately. Out of the three logistic regression models (table 2) that adjusted for area of residence, maternal age and maternal qualification, the latter (model 3) led to the greatest reduction in the parameter estimates associated with housing tenure, while the former (model 1) to the smallest.

**Table 2:** Unadjusted and partially adjusted ORs of lower respiratory tract infection hospital admissions in children aged <2 years by housing tenure

	Housing tenure	Unadjusted	Model 1	Model 2	Model 3
		OR (95% CI)	OR (95% CI)	OR (95% CI)	OR (95% CI)
Cohort 1 (n=126 187)	Owned (ref)	1.00	1.00	1.00	1.00
	Social rented	1.56 (1.46 to 1.66)	1.56 (1.47 to 1.66)	1.50 (1.40 to 1.61)	1.40 (1.31 to 1.50)
	Private rented	1.31 (1.17 to 1.46)	1.31 (1.17 to 1.47)	1.27 (1.14 to 1.43)	1.24 (1.11 to 1.39)
	Rent-free	1.69 (1.49 to 1.92)	1.71 (1.51 to 1.94)	1.63 (1.43 to 1.86)	1.52 (1.33 to 1.73)
Cohort 2 (n=135 646)	Owned (ref)	1.00	1.00	1.00	1.00
	Social rented	1.39 (1.32 to 1.47)	1.39 (1.32 to 1.47)	1.36 (1.28 to 1.44)	1.24 (1.17 to 1.32)
	Private rented	1.22 (1.14 to 1.30)	1.21 (1.14 to 1.30)	1.19 (1.11 to 1.27)	1.15 (1.08 to 1.23)
	Rent-free	1.07 (0.83 to 1.40)	1.09 (0.84 to 1.42)	1.06 (0.82 to 1.38)	1.05 (0.81 to 1.37)

Model 1: adjusted for area of residence (urban/rural); model 2: adjusted for maternal age; model 3: adjusted for highest maternal qualification

**Figure 3:**
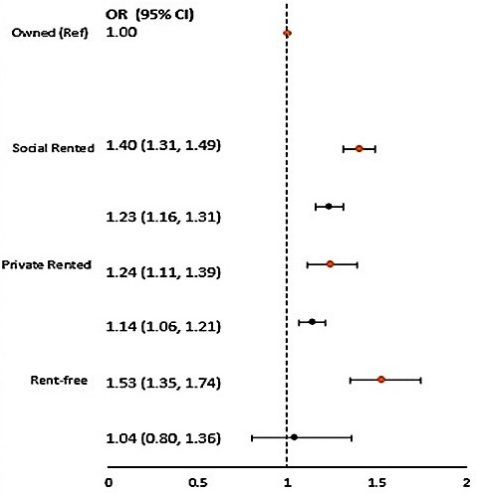
Maximally adjusted ORs and 95% CIs of lower respiratory tract infection admissions by housing tenure (C1, n=126 187; C2, n=135 646). Models adjusted for area of residence (urban/rural), maternal age and maternal highest qualifications. Orange indicates cohort 1; black indicates cohort 2.

In both cohorts, non-owned housing (social rented, private rented, rent-free housing) was associated with higher odds of LRTI admission compared with owned in the model that jointly adjusted for area of residence, maternal age and maternal highest qualifications (figure 3); in C1, the odds of early-childhood LRTI was 40% (95% CI 31% to 49%) higher for children living in social rented homes, 24% (11% to 39%) higher for children living in private rented homes and 53% (35% to 74%) higher for those in rent-free housing. In C2, the odds were 23% (95% CI 16% to 31%) higher for children living in social rented housing and 14% (6% to 21%) higher in private rented housing. For rent-free households, we did not see a statistically significant difference in odds compared with that in owned households (OR 1.04, 95% CI 0.80 to 1.36). The risk of early-childhood LRTI for children living in non-owned housing was more attenuated in C2: the OR for LRTI admission was reduced by 17% for social renters and by 10% for private renters in C2 compared with those in C1. We found no evidence of statistically significant effect modification of the tenure–LRTI association by parity or maternal smoking in pregnancy (results not shown).

## Discussion

We found a higher odds of LRTI admission among children living in rented accommodation in the early 2000s and 2010s, although the association appeared to be attenuated over time. The association between growing up in rented accommodation and odds of LRTI admission was not modified by factors such as parity or maternal pregnancy smoking history.

Our use of linked, national Census, vital statistics and administrative hospital data for all children born in Scotland reduces the risk of selection bias and minimises loss to follow-up. With a sample size of nearly 270 000 children, risks were examined in small subgroups, such as those living in rent-free housing. By using linked maternal Census data from two distinct time periods, we were able to observe changes in both family residential circumstances and LRTI admissions.

Like all studies using administrative data, there is a possibility of bias introduced by linkage errors. We noted a slightly higher prevalence of LRTI admission among children not linked to a maternal Census record, potentially indicating selection bias which could lead to an underestimation of the association between housing tenure and LRTI admission. We also lacked information on childcare attendance, a known risk factor for LRTI admission in children under age 2 years, which may have reduced the time spent in the home environment in C2.^24^ As our cohort was formed of children born in Scotland, we were unable to capture children who arrived in Scotland in the early weeks and months following their birth. Further, as with any observational study residual confounding can remain. Future research could explore whether early-life vulnerabilities, such as low birth weight or young gestational age, mediate the relationship between housing ownership status and LRTI admission as these early factors are likely to be associated with disadvantage and home ownership-recognised risk factors for LRTI.^25^ Finally, although we used the most recent Census data available at the time when data were requested, it would be of interest to update the study using 2021 data to examine possible temporal trends in the housing tenure-LRTI admission associations.

We identified an increase in LRTI admission risk, from 4.0% (C1) to 5.3% (C2), which is consistent with UK studies spanning a similar period.^10^ ^11^ Comparable studies of this size, of housing ownership status and paediatric LRTI admissions, however, are scarce. One smaller New Zealand study of LRTI admissions (children <5 years old) reported similar results (crude model), with children in non-owned housing having a 41% higher risk of admission.^26^ In models adjusted for maternal and child factors, this association no longer remained statistically significant.

We found that even after adjusting for socio-economic status, children living in rented accommodation were more likely to have an LRTI admission. This is of concern, considering that across Europe the number of families with children residing in owned homes is decreasing while the number living in private rented homes is growing.^19^ In a European study spanning 31 countries, renters in all the 31 countries had higher-than-average levels of ‘housing precariousness’ (based on affordability, security, quality, facilities and access to essential services).^27^ It is private renters who are most likely to experience multiple adverse housing circumstances, such as overcrowding, non-decent and unaffordable housing,^28^ all of which are associated either directly^29 30^ or indirectly^31^ with LRTI occurrence or severity. Mould and damp, which is more common in rental accommodation in the UK,^16^ is of particular concern due to its association with LRTI occurrence^18^ and hospitalisation^32^ in children.

Our research highlights the early-life respiratory health inequalities associated with housing ownership status. Considering the existing evidence that retrofitting existing homes or building high-quality new ones can reduce the burden of respiratory diseases,^33^ there is an urgent need to (1) undertake more research to understand which mechanisms underlie the association between rental housing and LRTI admissions and (2) link housing tenure and housing quality records to health records and use these data at a local level to inform place-based public health approaches to prevent LRTI admissions.

## Conclusion

Children living in social and private rented housing (compared with owned) have a higher risk of LRTI hospital admission in the first 2 years of life. Although the mechanism of how housing tenure impacts early LRTIs is still unknown, families in rental housing may benefit most from interventions to lower LRTI incidence, such as vaccination programmes. Further research is needed to elucidate the pathways from housing tenure to LRTI admission, especially the role of specific adverse housing conditions. Updating our analysis with the most recent Census information could further enhance its relevance.

## Supplementary material

10.1136/bmjph-2024-001965 online supplemental file 1

## Data Availability

Data may be obtained from a third party and are not publicly available.
