# Supplementary material for: Housing tenure and early-life acute lower respiratory tract infection admissions in two national Scottish birth cohorts
Source: BMJ Public Health. 2025 Jul 17;3(2):e001965. doi: 10.1136/bmjph-2024-001965 (PMC12273148; doi:10.1136/bmjph-2024-001965)
Supplement: online supplemental file 1 [file bmjph-3-2-s001.docx]

# Supplementary Materials

## **Supplementary Table 1 Data sources included variables and their descriptions.**

| **Type** | **Source** | **Source Description** | **Variables** |
| --- | --- | --- | --- |
| **Birth Records** | National Records of Scotland (NRS)  Vital event (birth) records. | Registrations of births, deaths, stillbirths, and deaths occurring in the first year of life. | Week of birth  Year of birth  Sex (baby) assigned at birth  Total births live and still (multiple/singleton)  Area of residence (UR8)*  Maternal country of origin (UK born/non-UK born)  Maternal country of residence (Scotland/England/other) |
|  | Scottish Birth Record | Patient level record of neonatal care, from 2002 onwards. | Week of birth |
| **Death Records** | National Records of Scotland (NRS)  Vital event (death) records | Deaths recorded (beyond the first year of life). | Week of death  Cause of death  Age in days |
| **Address Records** | Community Health Index Database | Individual patient identifier (assigned to all NHS patients) providing longitudinal data on migration history. | Transfer out of Scotland (yes/no)  Date of transfer |
| **Maternity/delivery records** | Scottish Morbidity Record 02  (SMR-02) | Patient level longitudinal hospital obstetric records for the mother. | Count of previous pregnancies (parity)**  Birth weight (<2,500g/2,500-3,499/3,500+grams)  Estimated gestational age: (pre-term< 37/ term37-42/post-term 41+ weeks).  Maternal age (<20/20-29/30-39/40+years)  Maternal pregnancy smoking history (no smoking in pregnancy/any smoking in pregnancy) |
| **Hospital admission records (child)** | Scottish Morbidity Record 01  (SMR**-**01) | Longitudinal hospital records for the child.  (General/acute, inpatient and day case dataset). | Admission date  Discharge date.  Length of stay  Admission type  Main condition (ICD-10) |
| **Maternal Census**  **(2001 & 2011)** | Decennial Census records (mother) | Resident-level social and demographic information.  N.B. Represents the entire population of Scotland. | Maternal highest qualification level***  Housing tenure (Owned/social rented/private rented/rent-free)  Accommodation type (detached/semi-detached/terraced/flat or maisonette/mobile or temporary accommodation)  Central heating type (no central heating/one or more) |

*Area of residence was derived from the *UR8* variable. (1) We assigned the classification closest to the child’s birth year, (2003-2004 version for births between 2000-2002), (2011-2012 version for births between 2010 and 2012). It is comprised of eight categories: 1, large urban areas; 2, other urban areas; 3, accessible small towns; 4, remote small towns; 5,very remote small towns;6, accessible rural areas; 7, remote rural areas;8, very remote rural areas. Categories 1-5 are classed as urban and 6-8 as rural areas.

**Count of previous completed pregnancies (parity) was consolidated into three categories: no previous completed pregnancies/one previous completed pregnancy/more than one previous completed pregnancies.

***Highest maternal qualification level was coded as: No qualifications/Level 1-Standard or equivalent/Level 2/Level 3/Level 4-First or higher degree/Not Applicable (<16 years old)

## **Supplementary Figure. 1 Graphical diagram of the presumed associations linking housing tenure with Lower Respiratory Tract Admission before the 2^nd^ birthday**


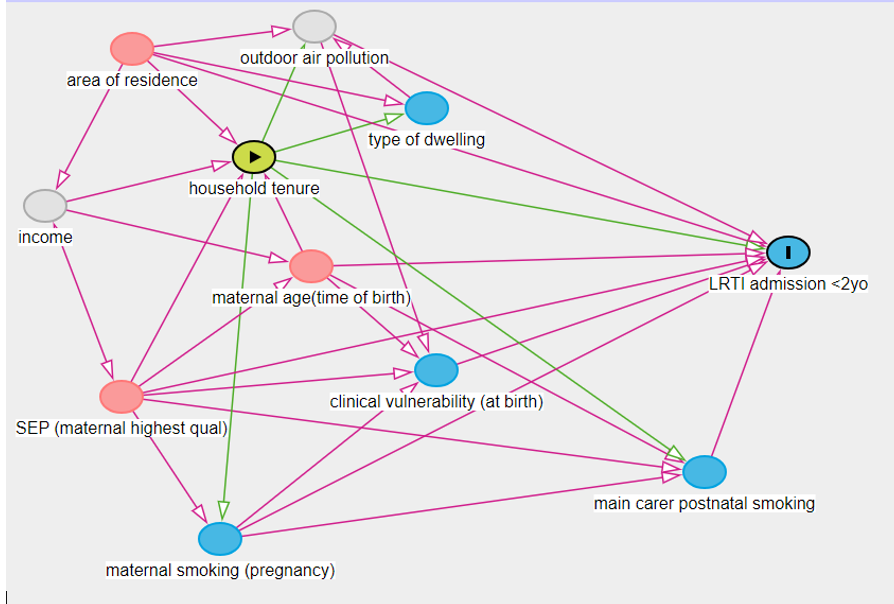


Diagram produced using Daggity

^[[1]](#footnote-2)^

The covariates which were adjusted for in the multivariable regression models (shown as pink nodes in this diagram) were selected as a minimal sufficient adjustment set for the presumed association linking housing tenure with LRTI admission, before the child’s second birthday.

## **Supplementary Table 2 Socio-demographic profile of the birth cohorts**

|  | **Cohort 1** | | **Cohort 2** | |
| --- | --- | --- | --- | --- |
|  | N | % | N | % |
|  | 130,739 | 100 | 139,203 | 100 |
| **CHILD CHARACTERISTICS** |  | | |  |
| **SEX** | | | | |
| Male | 66,824 | 51.1 | 71,266 | 51.2 |
| Female | 63,915 | 48.9 | 67,937 | 48.8 |
| Missing | 0 | 0 | 0 | 0 |
| **PARITY** | | | | |
| 0 | 55,930 | 42.8 | 59,046 | 42.4 |
| 1 | 44,939 | 34.3 | 48,296 | 34.7 |
| >1 | 24,807 | 19.0 | 27,378 | 19.7 |
| Missing | 5,063 | 3.9 | 4,483 | 3.2 |
| **BIRTHWEIGHT (g)** |  |  |  |  |
| <2,500 | 6,634 | 5.1 | 6,231 | 4.5 |
| 2,500-3,499 | 64,706 | 48.7 | 65,575 | 47.1 |
| 3,500+ | 55,797 | 42.7 | 63,835 | 45.9 |
| Missing | 4,602 | 3.5 | 3,562 | 2.5 |
| **ESTIMATED GESTATIONAL AGE (weeks)** | | | | |
| Pre-Term <37 | 7,098 | 5.4 | 7,178 | 5.1 |
| Term 37-41 | 86,123 | 65.9 | 93,811 | 67.4 |
| Post-Term 41+ | 32,928 | 25.2 | 34,629 | 24.9 |
| Missing | 4,590 | 3.5 | 3,585 | 2.6 |
| **MATERNAL CHARACTERISTICS** |  |  |  |  |
| **COUNTRY OF ORIGIN** |  |  |  |  |
| UK | 122,816 | 93.9 | 121,410 | 87.2 |
| Non-UK | 7,921 | 6.1 | 17,782 | 12.8 |
| Missing | 2 | 0 | 11 | 0 |
| **AGE (YEARS)** |  |  |  |  |
| <20 | 9,750 | 7.5 | 7,596 | 5.5 |
| 20-29 | 54,040 | 41.3 | 59,953 | 43.1 |
| 30-39 | 59,269 | 45.3 | 62,972 | 45.2 |
| >40 | 3,128 | 2.4 | 5,176 | 3.7 |
| Missing | 4,552 | 3.5 | 3,506 | 2.5 |
| **HIGHEST QUALIFICATION** | | | | |
| No qualifications | 18,118 | 13.9 | 10,930 | 7.8 |
| Level 1 Standard or equivalent | 45,692 | 35.0 | 37,678 | 27.1 |
| Level 2 Higher/advanced level | 22,855 | 17.5 | 19,560 | 14.1 |
| Level 3 SVQ Level 4 or equivalent | 12,617 | 9.7 | 19,112 | 13.7 |
| Level 4 First or higher degree | 30,504 | 23.3 | 51,423 | 36.9 |
| NA (No code required) or missing* | 953 | 0.6 | 500 | 0.4 |
| **TOBACCO SMOKING (PREGNANCY)** | | | | |
| No | 89,807 | 68.7 | 105,774 | 76.0 |
| Yes | 30,973 | 23.7 | 22,230 | 16.0 |
| Missing | 9,959 | 7.6 | 11,199 | 8.0 |
| **RESIDENTIAL CHARACTERISTICS** |  |  |  |  |
| **AREA OF RESIDENCE** |  |  |  |  |
| Urban | 107,738 | 82.4 | 117,652 | 84.6 |
| Rural | 23,001 | 17.6 | 21,499 | 15.4 |
| Missing | 0 | 0 | 52 | 0 |
| **HOUSEHOLD TENURE** |  |  |  |  |
| Owned | 84,258 | 64.5 | 79,401 | 57.0 |
| Social rented | 32,750 | 25.1 | 33,839 | 24.3 |
| Private rented | 8,563 | 6.5 | 24,660 | 17.8 |
| Rent Free | 5,168 | 3.9 | 1,303 | 0.9 |
| Missing | 0 | 0 | 0 | 0 |
| **ACCOMODATION TYPE** |  |  |  |  |
| Detached | 26,546 | 20.3 | 27,269 | 19.6 |
| Semi-detached | 32,824 | 25.1 | 32,987 | 23.7 |
| Terraced | 27,387 | 21.0 | 28,165 | 20.2 |
| Flat/maisonette/mobile/temp | 43,982 | 33.6 | 50,782 | 36.5 |
| Missing | 0 | 0 | 0 | 0 |
| **HEATING TYPE** |  |  |  |  |
| No central heating  One or more types of heating  Missing | 6,272 | 4.8 | 1,861 | 1.3 |
|  | 124,467 | 95.2 | 137,342 | 98.7 |
|  | 0 | 0 | 0 | 0 |

*For Highest Qualification the not applicable (NA) group was combined with the ‘No qualifications’ group due to small values

## **Supplementary Table 3 Maternal Census linkage quality**

|  | **Cohort 1** | | **Cohort 2** | |
| --- | --- | --- | --- | --- |
|  | **Census record**  *N (%)* | **No Census record** *N (%)* | **Census record**  *N (%)* | **No Census record**  *N (%)* |
| All | 133,392 (88.1) | 18,040 (11.9) | 142,108 (84.0) | 26,968 (16.0) |
| LRTI Admission (<2yo) | 5,305 (4.0) | 795 (4.4) | 7,446 (5.2) | 1,539 (5.7) |
| Low birthweight (<2,500) | 6,993 (5.2) | 1,272 (7.0) | 6,485 (4.6) | 1,757 (6.5) |
| Pre-term birth (Weeks) | 7,399 (5.5) | 1,168 (6.5) | 7,399 (5.2) | 1,652 (6.1) |
| Parity (>1 sibling) | 25,282 (18.9) | 3,443 (19.1) | 27,884 (19.6) | 6,419 (23.8) |
| Maternal age (<20 years) | 9,949 (7.5) | 2,469 (13.7) | 7,755 (5.5) | 2,225 (8.2) |

| **Supplementary Table 4 Child and family characteristics stratified by housing tenure (based on a sub-sample with complete records for all the adjustment variables)** | | | | | | | | | | | | | | | | | | | | | |
| --- | --- | --- | --- | --- | --- | --- | --- | --- | --- | --- | --- | --- | --- | --- | --- | --- | --- | --- | --- | --- | --- |
|  | **Cohort 1**  **Births 2000-2002 n=126,187** | | | | | | | | | | **Cohort 2**  **Births 2010-2012 n=135,646** | | | | | | | | | | |
| **ALL**  **LRTI ADMISSION** | **Owned** | | **Social Rent** | | **Private Rent** | | | **Rent Free** | | | **Owned** | | **Social Rent** | | | **Private Rent** | | | **Rent Free** | | |
|  | **N (%)** | | **N (%)** | | **N (%)** | | | **N (%)** | | | **N (%)** | | **N (%)** | | | **N(%)** | | | **N (%)** | | |
|  | 81,451 (64.5) | | 31,569 (25.0) | | 8,173 (6.5) | | | 4,994 (4.0) | | | 77,312 (57.0) | | 33,064 (24.4) | | | 24,011 (17.7) | | | 1,259 (0.9) | | |
|  | 2,775 (3.4) | | 1,643 (5.2) | | 360 (4.4) | | | 281 (5.6) | | | 3,609 (4.7) | | 2,111 (6.4) | | | 1,350 (5.6) | | | 63 (5.0) | | |
| **CHILD** | | | | | | | | | | | | | | | | | | | | | |
| **PARITY (Number of siblings)** | | | | | | | | | | | | | | | | | | | | | |
| 0 | 38,166 (46.9) | | 12,091 (38.3) | | 3,816 (46.7) | | | 1,857 (37.2) | | | 35,091 (45.4) | | 12,240 (37.0) | | | 11,121 (46.3) | | | 572 (45.4) | | |
| 1 | 30,202 (37.1) | | 10,426 (33.0) | | 2,507 (30.7) | | | 1,803 (36.1) | | | 28,853 (37.3) | | 11,039 (33.4) | | | 7,931 (33.1) | | | 452 (35.9) | | |
| >1 | 12,758 (15.6) | | 8,935 (28.3) | | 1,798 (22.0) | | | 1,316 (26.3) | | | 12,776 (16.5) | | 9,561 (28.9) | | | 4,807 (20.0) | | | 227 (18.0) | | |
| Missing | 325 (0.4) | | 117 (0.4) | | 52 (0.6) | | | 18 (0.4) | | | 592 (0.8) | | 224 (0.7) | | | 152 (0.6) | | | 8 (0.7) | | |
| **BIRTHWEIGHT (g)** | | | | | | | | | | | | | | | | | | | | | |
| Missing | 3,380 (4.1) | | 2,406 (7.6) | | 469 (5.7) | | | 379 (7.6) | | | 2,797 (3.6) | | 2,219 (6.7) | | | 1,169 (4.9) | | | 46 (3.7) | | |
| 2,500-3,499 | 39,247 (48.2) | | 17,428 (55.2) | | 4,331 (53.0) | | | 2,700 (54.1) | | | 35,555 (46.0) | | 17,455 (52.8) | | | 11,978 (49.9) | | | 563 (44.7) | | |
| 3,500 + | 38,791 (47.6) | | 11,724 (37.1) | | 3,369 (41.2) | | | 1,912 (38.3) | | | 38,928 (50.3) | | 13,378 (40.5) | | | 10,852 (45.2) | | | 650 (51.6) | | |
| Missing | 33 (0.1) | | 11 (0.1) | | 4 (0.1) | | | 3.0 (0.1) | | | 32 (0.1) | | 12 (<0.1) | | | 12 (<0.1) | | | 0 (0.0) | | |
| **GESTATIONAL AGE (Weeks)** | | | | | | | | | | | | | | | | | | | | | |
| Pre-Term <37 | 4,075 (5.0) | | 2,200 (7.0) | | 475 (5.8) | | | 348 (7.0) | | | 3,689 (4.7) | | 2,146 (6.5) | | | 1,293 (5.4) | | | 49 (3.9) | | |
| Term 37-41 | 55,620 (68.3) | | 21,500 (68.1) | | 5,538 (67.8) | | | 3,464 (69.4) | | | 53,240 (68.9) | | 23,192 (70.1) | | | 16,479 (68.6) | | | 866 (68.8) | | |
| Post-Term 41+ | 21,736 (26.7) | | 7,856 (24.9) | | 2,158 (26.4) | | | 1,178 (23.6) | | | 20,338 (26.3) | | 7,705 (23.3) | | | 6,226 (25.9) | | | 344 (27.3) | | |
| Missing | 20 (0.1) | | 13 (<0.1) | | 2 (<0.1) | | | 4.0 (0.1) | | | 45 (0.1) | | 21 (0.1) | | | 13. (0.1) | | | 0 (0.0) | | |
| **MATERNAL** |  | | | | | | | | | | | | | | | | | | | | |
| **MATERNAL AGE (Years)** |  | | | | | | | | | | | | | | | | | | | | |
| <20 | 2,765 (3.4) | | 5,129 (16.2) | | 847 (10.3) | | | 1,009 (20.2) | | | 1,803 (2.3) | | 4,227 (12.8) | | | 1,517 (6.3) | | | 49 (3.9) | | |
| 20-29 | 29,816 (36.6) | | 17,352 (55.0) | | 4,223 (51.7) | | | 2,649 (53.0) | | | 25,511 (33.0) | | 19,523 (59.0) | | | 14,280 (59.5) | | | 617 (49.0) | | |
| 30-39 | 46,474 (57.1) | | 8,590 (27.2) | | 2,941 (36.0) | | | 1,264 (25.3) | | | 45,998 (59.5) | | 8,660 (26.2) | | | 7,737 (32.2) | | | 550 (43.7) | | |
| >40 | 2,396 (2.9) | | 498 (1.6) | | 162 (2.0) | | | 72 (1.5) | | | 4,000 (5.2) | | 654 (2.0) | | | 477 (2.0) | | | 43 (3.4) | | |
| Missing | 0 (0.0) | | 0 (0.0) | | 1. (0.0) | | | 0 (0.0) | | | 0 (0.0) | | 0 (0.0) | | | 0 (0.0) | | | 0 (0.0) | | |
| **HIGHEST QUALIFICATION** |  | | | | | | | | | | | | | | | | | | | | |
| No qualifications/NA* | 5,637 (6.9) | | 9,596 (30.4) | | 1,442 (17.7) | | | 1,568 (31.4) | | | 1,975 (2.5) | | 6,638 (20.1) | | | 2,417 (10.1) | | | 102 (8.1) | | |
| Level 1 Standard | 24,310 (29.9) | | 14,908 (47.2) | | 2,954 (36.1) | | | 2,177 (43.6) | | | 13,574 (17.6) | | 15,504 (46.9) | | | 7,587 (31.6) | | | 251 (19.9) | | |
| Level 2 Higher/advanced | 16,326 (20.0) | | 3,921(12.4) | | 1,343 (16.4) | | | 540 (10.8) | | | 11,278 (14.6) | | 4,246 (12.8) | | | 3,390 (14.1) | | | 202 (16.1) | | |
| Level 3 SVQ Level 4 | 9,227 (11.3) | | 2,015 (6.4) | | 706 (8.6) | | | 258 (5.2) | | | 11,453 (14.8) | | 3,723 (11.3) | | | 3,338 (13.9) | | | 174 (13.8) | | |
| Level 4 First/higher degree | 25,951 (31.9) | | 1,129 (3.6) | | 1,728 (21.2) | | | 451 (9.0) | | | 39,032 (50.5) | | 2,953 (8.9) | | | 7,279 (30.3) | | | 530 (42.1) | | |
| Missing | 0 (0.0) | | 0 (0.0) | | 1. (0.0) | | | 0 (0.0) | | | 0 (0.0) | | 0 (0.0) | | | 0 (0.0) | | | 0 (0.0) | | |
| **COUNTRY OF ORIGIN** |  |  |  |  | |  |  | |  |  |  |  | |  |  | |  |  | |  |  |
| UK Born | 76,147 (93.5) | | 30,537 (96.7) | | 7,179 (87.8) | | | 4,741 (94.9) | | | 69,250 (89.6) | | 29,681 (89.8) | | | 18,421 (76.7) | | | 998 (79.3) | | |
| Non-UK born | 5,303 (6.5) | | 1,032 (3.3) | | 993 (12.2) | | | 253 (5.1) | | | 8,056 (10.4) | | 3,381 (10.2) | | | 5,588 (23.3) | | | 261 (20.7) | | |
| Missing | 1 (<0.1) | | 0 (0.0) | | 1 (<0.1) | | | 0 (0.0) | | | 6 (<0.1) | | 2 (<0.1) | | | 2 (<0.1) | | | 0 (0.0) | | |
| **TOBACCO SMOKING**  **(DURING PREGNANCY)** |  | | | | | | | | | | | | | | | | | | | | |
| No | 67,018 (82.3) | | 15,077 (47.8) | | 5,299 (64.8) | | | 2,412 (48.3) | | | 67,680 (87.5) | | 19,156 (57.9) | | | 17,844 (74.3) | | | 1,049 (83.3) | | |
| Yes | 11,400 (14.0) | | 14,760 (46.7) | | 2,534 (31.0) | | | 2,279 (45.6) | | | 5,472 (7.1) | | 11,863 (35.9) | | | 4,763 (19.8) | | | 126 (10.0) | | |
| Missing | 3,033 (3.7) | | 1,732 (5.5) | | 340 (4.2) | | | 303 (6.1) | | | 4,160 (5.4) | | 2,045 (6.2) | | | 1,404 (5.9) | | | 84 (6.7) | | |
|  |  | |  | |  | | |  | | |  | |  | | |  | | |  | | |
|  |  | |  | |  | | |  | | |  | |  | | |  | | |  | | |
|  |  | |  | |  | | |  | | |  | |  | | |  | | |  | | |

| **RESIDENTIAL** | | | | | | | | | |
| --- | --- | --- | --- | --- | --- | --- | --- | --- | --- |
| **AREA OF RESIDENCE URBAN/RURAL** |  | | | | | | | | |
| Urban | 66,356 (81.5) | 27,939 (88.5) | 5,809 (71.0) | 3,936 (78.8) | 64,209 (83.1) | 29,567 (89.4) | 20,449 (85.2) | 604 (48.0) |  |
| Rural | 15,095 (18.5) | 3,630 (11.5) | 2,364 (29.0) | 1,058 (21.2) | 13,103 (16.9) | 3,497 (10.6) | 3,562 (14.8) | 655 (52.0) |  |
| Missing | 0 (0.0) | 0 (0.0) | 0 (0.0) | 0 (0.0) | 0 (0.0) | 0 (0.0) | 0 (0.0) | 0 (0.0) |  |
| **HEATING TYPE** |  |  |  |  |  |  |  |  |  |
| No central heating | 2,455 (3.0) | 1,952 (6.2) | 1,141 (14.0) | 442 (8.8) | 791 (1.0) | 319 (1.0) | 650 (2.7) | 51 (4.0) |  |
| One or more types | 78,996 (97.0) | 39,617 (93.8) | 7,032 (86.0) | 4,552 (91.2) | 76,521 (99.0) | 32,745 (99.0) | 23,361 (97.3) | 1,208 (96.0) |  |
| Missing | 0 (0.0) | 0 (0.0) | 0 (0.0) | 0 (0.0) | 0 (0.0) | 0 (0.0) | 0 (0.0) | 0 (0.0) |  |

*The Not applicable (NA) group was combined with the ‘No qualifications’ group due to small values

1. Johannes Textor, Benito van der Zander, Mark K. Gilthorpe, Maciej Liskiewicz, George T.H. Ellison.
   [Robust causal inference using directed acyclic graphs: the R package 'dagitty'.](http://dx.doi.org/10.1093/ije/dyw341)
   *International Journal of Epidemiology* 45(6):1887-1894, 2016. [↑](#footnote-ref-2)
